# Metformin in patients with and without diabetes: a paradigm shift in cardiovascular disease management

**DOI:** 10.1186/s12933-019-0860-y

**Published:** 2019-04-27

**Authors:** Fei Luo, Avash Das, Jingfei Chen, Panyun Wu, Xiangping Li, Zhenfei Fang

**Affiliations:** 1 Department of Cardiovascular Medicine, The Second Xiangya Hospital, Central South University, Changsha, 410011 Hunan China; 2 Departments of Molecular Genetics, University of Texas Southwestern Medical Center, Dallas, TX 75390 USA; 3 Department of Obstetrics and Gynecology, Xiangya Hospital, Central South University, Changsha, 410011 Hunan China

**Keywords:** Metformin, Diabetes mellitus, Coronary artery disease, Atherosclerosis

## Abstract

With an increasing global burden of coronary artery disease (CAD), early detection and timely management of risk factors are crucial to reduce morbidity and mortality in such patients. Diabetes mellitus (DM) is considered an independent risk factor for the development of CAD. Metformin, an anti-diabetic drug, has been shown in pre-clinical and clinical studies, to lower the cardiovascular events in the DM patients. Growing evidence suggests that metformin has a protective effect on coronary artery beyond its hypoglycemic effects. Given its global availability, route of administration and cost, metformin provides an alternate/additional therapeutic option for primary and secondary prevention of CAD in DM and non-diabetics alike. Future prospective cohort-based studies and randomized clinical trials are needed to identify ‘at-risk’ population who may potentially benefit from metformin.

## Introduction

**Fig. 1:**
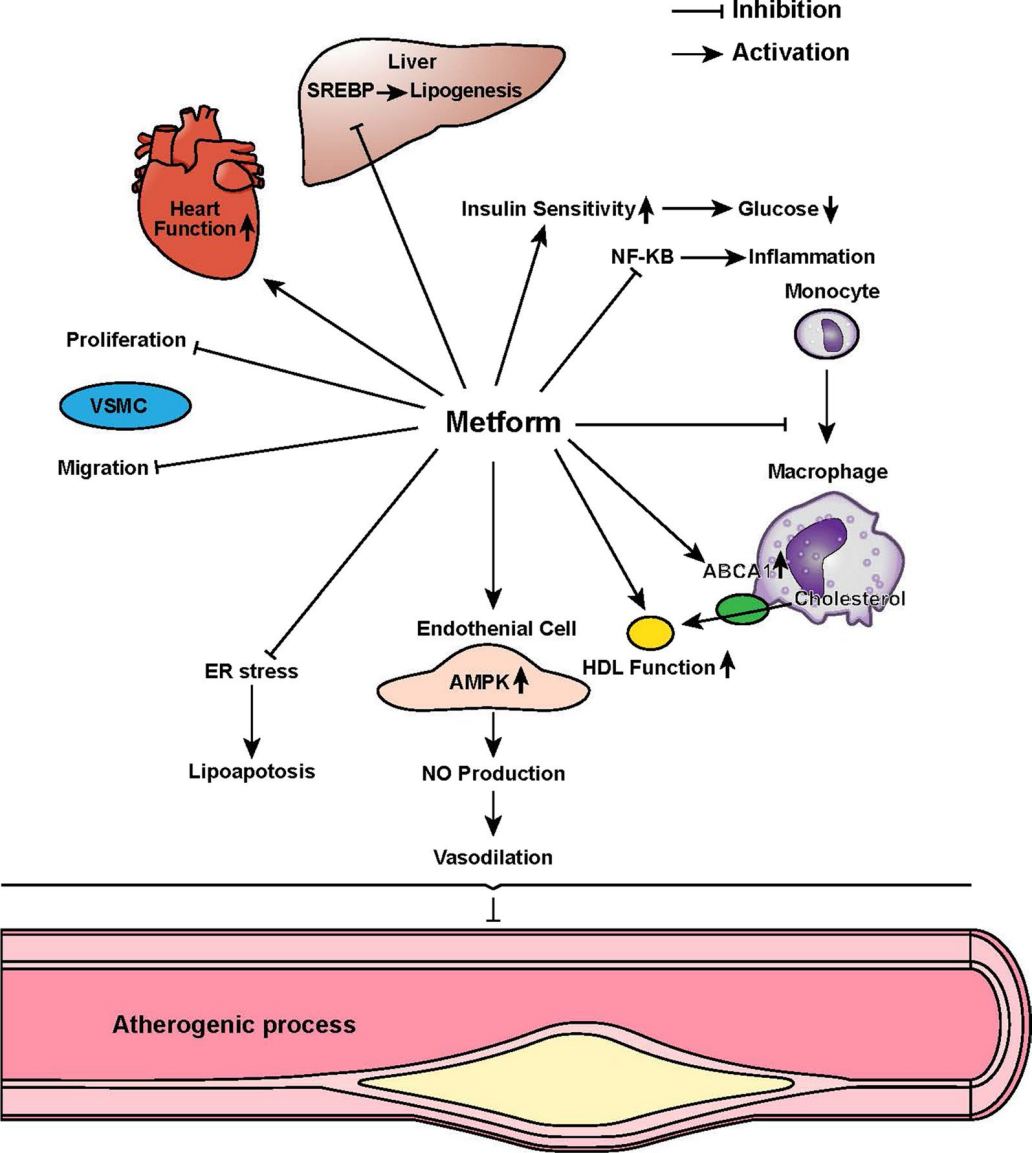
Beneficial pleiotropic effects of metformin on cardiovascular and the potential mechanisms. *SREBP* sterol regulatory element-binding proteins, *ABCA1* ATP-binding cassette transporter A1, *AMPK* AMP-activated protein kinase, *NO* nitric oxide, *ER* endoplasmic reticulum, *HDL* high-density lipoprotein

Coronary artery disease (CAD) is among the leading cause of mortality and morbidity worldwide and puts an enormous economic burden in the society [1]. There are multiple risk factors of CAD like tobacco, obesity, hypertension and high blood cholesterol [2]. Diabetes mellitus (DM) is considered to be an independent risk factor in the development of CAD [3]. Macrovascular complications of DM, involving the cardiovascular system, constitute the leading cause of death in longstanding diabetics [4–6]. Metformin, a prominent biguanide class of drugs, controls the level of blood glucose in the body through lowering of the peripheral insulin resistance and concurrent decrease in intestinal absorption of glucose [7]. Current treatment guidelines recommend the use of metformin as a first-line therapy for patients with DM [8]. While primarily used as an anti-diabetic drug, other pleiotropic effects of metformin remained largely unexplored. Burgeoning evidence points towards the cardioprotective effects of metformin in its improvement of cardiovascular outcomes in patients with well-defined risk factors. Although clinical trials conducted on diabetic patients clearly demonstrated the therapeutic potential of metformin in reducing cardiovascular mortality and morbidity in DM patients [9], their beneficial effects in non-diabetic patients remain unclear. The cardioprotective effect of metformin in DM patients can be attributed to its anti-atherosclerotic property. In this article, we review the existing literature reporting the anti-atherosclerotic property of metformin in the cardiovascular space (Fig. 1).

## Cardiovascular effects of metformin: clinical scenario

### Metformin in diabetes mellitus (DM)

Type 2 diabetes mellitus (T2DM) is considered as an independent risk factor for the development of CAD [10]. Therefore, tight blood glucose control is critical to limit the mortality and morbidity from CVD in T2DM patients. Metformin, a first line anti-diabetic drug, has been reported to reduce major cardiovascular events associated with atherosclerotic cardiovascular disease (ASCVD) in T2DM patients or improve the surrogate endpoints of ASCVD such as carotid intima-media thickness (CIMT). A landmark study in this area was the UK Protective Diabetes Study (UKPDS) [11], which randomized 1704 overweight (> 120% ideal body weight) patients with newly diagnosed T2DM to receive conventional treatment with diet alone in one trial arm or either metformin, sulphonylurea, or insulin in the other. After a median follow up of 10.7 years, the metformin group had a 36% lower all-cause mortality (*P* = 0.011) and a concurrent a 39% lower risk (*P* = 0.011) in the incidence of myocardial infarction than the conventional treatment group but did not differ significantly from the other intensive glucose control treatment group. However, in a combined analysis of a supplementary of the same clinical trial, where 537 non-overweight and overweight patients with uncontrolled plasma blood glucose (6.1–15.0 mmol/L) were treated with sulfonylureas with and without metformin, the effect of metformin on cardiovascular outcomes was not statistically significant. A possible explanation of this phenomenon could be related to the beneficial effect of tight glycemic control from metformin that prevented future cardiovascular consequences. Subsequent follow-up study of 10 years after UKPDS, however, reported continuous risk reduction of myocardial infarction (33%, *P* = 0.005) in patients treated with metformin despite no changes in the glycated hemoglobin (HbA1c) levels [12]. Since no new glucose-lowering therapy was introduced in the study cohort during this period, the results highlight the beneficial cardiovascular effects of metformin. This effect of metformin was particularly pronounced among overweight patients after a long duration of follow-up. In a continued effort to study the add-on effects of metformin on macrovascular or microvascular disease in insulin-treated T2DM patients, Kooy et al. [13] randomized 390 T2DM patients, with a mean age of 53 years, to receive metformin (850 mg/day) or placebo and followed them for 4.3 years. The results show that metformin treatment significantly improved the macrovascular end point compared with placebo (HR 0.61, 95% CI 0.40–0.94, *P* = 0.02), which could not be explained solely by the difference in weight and metformin-associated changes in metabolic or hemodynamic variables, such as HbA1C level [13]. Further study by Katakami et al. [14] in a cohort of 118 patients with T2DM who were randomized to receive glibenclamide (n = 59), gliclazide (n = 30), glibenclamide plus metformin (n = 29), with a median follow-up duration of 3 years, also showed that the CIMT in glibenclamide plus metformin group was significantly smaller than that in the glibenclamide and gliclazide groups in univariate and multivariate regression analysis (P < 0.05). A meta-analysis of 35 clinical RCTs confirmed the cardiovascular benefits of metformin in comparison to placebo in younger population followed over a long duration [15]. Taken together, these observations strengthen the rationale behind the use of metformin in ‘at-risk’ population (T2DM patients) from a younger age to decrease cardiovascular events.

A seminal study in the field, The study on the prognosis and effect of antidiabetic drugs on type 2 diabetes mellitus with coronary artery disease (SPREAD-DIMCAD) was conducted to evaluate the major cardiovascular events and mortality among type 2 diabetic patients with CAD after their treatment with glipizide or metformin [16]. Among the 304 T2DM patients enrolled for the RCT who were followed up for 5 years, the metformin group showed a significantly lower cardiovascular endpoint (recurrent cardiovascular events, including nonfatal myocardial infarction, nonfatal stroke or arterial revascularization by percutaneous transluminal coronary angioplasty (PTCA) or by coronary artery bypass graft, death from a cardiovascular cause) than the glipizide group (HR 0.54, 95% CI 0.30–0.90, *P* = 0.026). However, the glycated hemoglobin values in the two groups were similar (7.0% vs 7.1%, P > 0.05) [16]. The results were ‘proof of concept’, suggesting the pleiotropic effects of metformin in the heart and blood vessels, independent of its glucose-lowering activity.

The REversing with MetfOrmin Vascular Adverse Lesions (REMOVAL) Trial is the largest and longest double-blind placebo-controlled RCT to evaluate cardiovascular effect of metformin in adults with type 1 DM (T1DM) with a median follow up duration of 5 years in patients with high cardiovascular risk (have ≥ 3 of 10 specified cardiovascular risk factors) [17]. In REMOVAL trial, 428 insulin-treated patients were randomly assigned to metformin and placebo. At a follow-up visit, atherosclerosis progression, measured by the averaged maximal CIMT, was significantly reduced with metformin (− 0.013 mm/year, − 0.024 to − 0.003; P = 0.0093) [18], indicating possible cardiovascular benefits, but warrants further investigation. However, the significant reduction in HbA1c (− 0.13%, 95% CI − 0.22 to − 0.037; P = 0.0060) over 3 years was more robust over the initial 3-month after the commencement of treatment (− 0.24%, − 0.34 to − 0.13; P < 0.0001) [18]. In the SPREAD-DIMCAD trial, the metformin group showed a significantly lower cardiovascular endpoint with similar HbA1c level, suggesting the metformin effect is independent of blood sugar level. However, in REMOVAL trial, the reduction of CIMT was associated with HbA1c reduction with metformin. Taken together, the cardioprotective properties of metformin appears to be a consequence of its atheroprotective effect of metformin. However, the conundrum, concerning the association of the atheroprotective effect of metformin with its anti-hyperglycemic effects in T1DM patients, still remains unanswered.

Recent evidence demonstrates the synergistic effects of co-administration of metformin with other drugs. Treatment with metformin alongside empagliflozin, a new sodium glucose cotransporter-2 (SGLT2) inhibitor, has significantly improved arterial stiffness compared to metformin alone in T1DM patients [19]. The effect was higher than either combination of glitazones or alpha-glucosidase inhibitors with metformin and was also associated with lower major adverse cardiovascular events (MACE) risk in comparison to sulphonylureas and metformin combinatorial treatment in T2DM patients [20]. Metformin with Saxagliptin has been shown to improve the endothelial dysfunction in early diabetics [21] and its combination with vildagliptin is poised as a viable alternative in the treatment of T2DM and CAD due to the lower rate of recurrent cardiovascular events, in part due to its anti-inflammatory property [22]. Even, metformin with ascorbic acid has been shown to be effective in reducing risks for diabetes-related long-term complications (including albumin/creatinine ratio) [23]. However, increased BMI in metformin-exposed children during intrauterine development might confer them a higher risk of developing cardiometabolic diseases later in their adulthood [24]. Recent evidence supports the use of liraglutide as a viable alternative to metformin for recent-onset T2DM in women during their child-bearing age to circumvent such clinical scenarios [25].

### Metformin in non-diabetics

Although the cardiovascular benefits with metformin are well-established in diabetic patients, their role in non-diabetic patients remains elusive. In a small randomized double-blind placebo-controlled study consisting of 33 non-diabetic women, it was shown that metformin can reduce myocardial ischemia in female patients with angina, compared to placebo [26]. However, a study by Hao et al. [9] consisting of 130 patients with dyslipidemia and obesity who were randomized to atorvastatin or atorvastatin plus metformin, showed atorvastatin combined with metformin was more effective than atorvastatin monotherapy group in improving the rate of obesity and subclinical inflammation. A subsequent study by Eduardo et al. [27] confirmed that metformin decreased the CIMT (− 0.1 mm, P = 0.04, vs − 0.02 mm, P = not significant) in comparison to control group in patients with metabolic syndrome, thereby indicating its role in cardioprotection.

However, recent studies have questioned the validity of the conclusion from prior studies. The Carotid Atherosclerosis: MEtformin for insulin ResistAnce (CAMERA) [28] study involving 173 non-diabetic patients with CAD who were on statin therapy, were assigned to either metformin or matching placebo. No improvement in CIMT was reported in the metformin group (slope difference 0.007 mm/year, P = 0.29) when compared with a placebo group, although HbA1c, insulin, and insulin resistance index decreased significantly in the metformin group (P < 0.05) [28]. The possible explanations for the conflicting outcomes in these studies can be attributed to the difference in baseline characteristics of the patients (a type of disease, age, taking other hypoglycemic and lipid-lowering drugs), study endpoints and follow-up duration of the individual clinical study. Whether metformin has a cardiovascular benefit in pre-diabetic patients is not clear. An area of active research, ongoing multicenter RCT Glucose Lowering in Non-diabetic Hyperglycemia Trial (GLINT, ISRCTN34875079) is currently enrolling non-diabetic patients for treatment with metformin to evaluate the incidence of cardiovascular death and non-fatal myocardial infarction events. The findings from this study will provide more insight in the prophylactic use of metformin in a similar cohorts.

## Cardiovascular effects of metformin: translational and pre-clinical evidence

The large cache of clinical data demonstrating the cardioprotective effects of metformin warrants further mechanistic insight. Possible explanations of the cardioprotective effects of metformin can be due to its pleiotropic effects in blood vessels including endothelial cells, and smooth muscle cells, blood lipid and chronic systemic inflammation.

### Anti-atherosclerotic effect of metformin

Data accrued over a period of 30 years, has shown that metformin can reduce the formation of atherosclerotic plaques in animals fed on a high cholesterol diet [29, 30]. Li et al. [30] used a high-cholesterol diet to induce atherosclerosis in rabbits and studied the anti-atherosclerotic effect of metformin. They demonstrated metformin significantly reduces atherosclerotic plaque with decreased serum high-sensitivity C-reactive protein with concurrent inhibition the of NF-κB pathway activation in the vascular wall. Recent studies have also found that metformin can reduce plaque formation in a high cholesterol diet-induced atherosclerotic Apolipoprotein E knockout (ApoE^−/−^) murine model [31]. Calcification of atherosclerotic plaque has been associated with plaque instability and acts a strong indicator of poor clinical cardiovascular outcomes in patients [15, 16]. Recently, Cai et al. [32] reported that metformin [100 mg/(kg day)] significantly reduced calcification of atherosclerotic plaque in ApoE^−/−^ mice fed on a high-fat diet, suggesting that metformin may also improve plaque stability. Our prior study had also confirmed the anti-atherosclerotic property in high-cholesterol fed rabbits [33].

### Vascular endothelial protection effect of metformin

Vascular endothelial dysfunction is the first step in atherosclerosis and one of the important pathological processes. Clinical studies suggest that metformin may significantly improve endothelium-dependent vasodilation in patients with T2DM and polycystic ovarian syndrome [34, 35]. In a preclinical experiment, metformin has been shown to increase NO-mediated vasodilation in endothelial cells in vitro [36]. Further studies have shown that it may increase NO production by activating AMPK pathway and thus improve vascular endothelial function [37]. Studies have also found that activation of AMPKα2 attenuates endoplasmic reticulum stress in vascular endothelial cells [38]. Metformin, being an agonist of AMPKα2, can activate AMP-activated protein kinase and protect human coronary artery endothelial cells against diabetic lipoapoptosis [39], suggesting an alternative mechanism of cardioprotective effect in the body.

### Metformin and vascular smooth muscle cells

Vascular smooth muscle cells (VSMCs) proliferation, migration and phenotype conversion involved in the development of atherosclerosis [40], and calcification of VSMCs in atherosclerotic plaque is closely related to plaque instability [41]. Studies found that the activation of AMPKα2 can inhibit the abnormal migration of VSMCs, delay the intimal thickening and increase the stability of atherosclerotic plaques [42, 43]. Recent animal studies have shown that metformin can reduce the formation of calcification in VSMCs in atherosclerotic plaque through the activation of the AMPK pathway [17, 53]. Therefore, metformin may play an anti-atherosclerotic role through AMPK-mediated VSMCs regulation.

### Metformin and blood lipids

LDL-C level is an important risk factor for atherosclerosis and every 38.7 mg/dL reduction of LDL-C results in a 20% reduction in cardiovascular events [44]. A possible explanation of the cardioprotective property of metformin can be explained by its effects on lowering LDL-C. An observational study reported that metformin reduces LDL-C in patients with T2DM by about 11.85 mg/dL (P < 0.05) [45]. However, multiple pre-clinical studies [18–20] have also reported that metformin reduce the aortic cholesterol deposition and atherosclerotic plaque formation in high cholesterol diet-induced atherosclerotic rabbit or murine models, without affecting their serum total cholesterol (TC) and LDL-C levels. Subsequently, three RCTs: the CAMERA study (baseline LDL-C level: about 108.4 mg/dL and 100% of the subjects had statins), the HOME study (baseline LDL-C level: about 137.0 mg/dL, 34% of the subjects used statins) and the SPREAD study (baseline LDL-C level of about 107.9 mg/dL and 62% of the subjects used statins) did not report any reduction of LDL-C levels with metformin [13, 16, 28]. The difference in clinical outcomes in various studies can be a consequence of the difference in baseline LDL-C levels and/or types of lipid-lowering drugs used. Not surprisingly, both HOME and SPREAD had documented the cardiovascular benefit of metformin, implying that LDL-C reduction may not be a primary contributor in its anti-atherogenic effects.

In contrast to LDL-C, high-density lipoprotein cholesterol (HDL-C) has a cardioprotective effect with HDL-C levels being inversely associated with cardiovascular events. Besides the HDL-C levels, the improvement of HDL cholesterol efflux has become a new target for the treatment of ASCVD in recent years. Previous studies indicated impaired HDL function may accelerate the development of atherosclerosis [46], and reduced HDL cholesterol efflux is associated with an increased risk of ASCVD [47]. Patients with diabetes are often associated with decreased HDL levels and impaired function [48, 49]. Of note, an RCT including 3070 people with impaired glucose tolerance reported that metformin treatment increases HDL-C levels, but the effect wears off after adjusting for body mass index weakened (P = 0.06) [50]. The HOME and CAMERA studies also yielded similar results [13, 28]. Matsuki et al. [51] also found that HDL-mediated cholesterol efflux was significantly reduced after human HDL was glycosylated, and HDL-mediated cholesterol efflux returned to normal levels after intervention with metformin in glycosylated HDL, indicating metformin may exert anti-atherosclerotic function by improving cholesterol reverse function of HDL.

Emerging studies have found that elevated triglyceride (TG) levels increase the risk of ASCVD and lower blood TG can reduce cardiovascular events [52–55]. In pre-clinical studies, metformin treatment (200 mg/kg/day) in mice for 4 weeks significantly reduced serum TG levels under high-fat diet (− 38%, P < 0.05) [56]. Further studies found that metformin did not affect the production and secretion of very low-density lipoprotein (VLDL) in the liver, but promoted the fatty acid oxidation of brown fat, which may be related to the activation of adenosine monophosphate-activated protein kinase (AMP-activated kinase, AMPK) pathway [56]. We have previously shown that metformin ameliorates obesity-associated hypertriglyceridemia in mice partly through the apolipoprotein A5 pathway [57]. Apolipoprotein A5 is a novel member of the apolipoprotein family, was reported to have a strong ability to decrease serum concentrations of TG [58]. Clinical trial, HOME (metformin dosage: 850 mg/day) showed no significant difference in TG levels between metformin and placebo (0.88 mg/dL, P = 0.82) [13]. CAMERA study (metformin dosage: 1000 mg/day) also found that metformin had no significant effect on TG level (− 7.08 mg/dL, P = 0.054). However, a systematic review analyzed 41 clinical studies and found only high doge of metformin (> 1700 mg/day) decreased plasma TG significantly [59]. The negative result may due to the lower dosage used in these trials. In all, we can infer that metformin, at higher dose, could regulate TG levels and HDL function which may contribute to the anti-atherosclerotic effect.

### Anti-inflammatory effects of metformin

The chronic inflammatory process leading to atherosclerosis is well-documented [60]. Previous studies showed that high-sensitivity C-reactive protein is an independent risk factor for cardiovascular disease [37]. Li et al. [30] found metformin treatment at a dose of 150 mg/kg for 16 weeks significantly reduced serum high-sensitivity C-reactive protein in high-cholesterol diet fed rabbits. A possible mechanism of this inverse correlation may be related to the inhibition of NF-κB in the vascular wall by metformin [30]. In an in vitro study, Isoda et al. [61] also found that metformin can inhibit the activation of NF-κB in endothelial cells and vascular smooth muscle cells in a concentration-dependent manner, thus inhibiting the effect of interleukin-1β-induced inflammatory cytokines secretion. The mechanism of action of metformin has also been shown to be closely related to the AMPK pathway [62]. Hattori et al. [63] found metformin inhibited the TNF-α-induced activation of NF-κB in a dose-dependent manner via activating AMPK, which was attenuated by siRNA knockdown of AMPKα1, providing a possible explanation for the anti-inflammatory effect may be related to AMPK pathway activation.

### Metformin and mononuclear macrophages

Atherosclerotic plaques mainly consist of lipid-rich foam cells deposited under the intima. Mononuclear macrophages migrate to the intima, phagocytose cholesterol-containing lipids through cell membrane surface scavenger receptors and transform into foam cells. In this process, the expressions of cholesterol efflux related receptors, such as ATP-binding cassette transporter A1 (ABCA1) and G1 are downregulated and the cholesterol efflux capacity decreased [64, 65]. Promoting the expression of ABCA1 and ABCG1 can potentially inhibit the conversion of monocyte-derived macrophages into foam cells which contributes to preventing the formation and progression of atherosclerotic plaque. In animal models, Vasamsetti et al. [66] found metformin inhibits angiotensin II-induced lipid deposition in macrophages and reduces the formation of atherosclerotic plaques. In vitro experiments found that metformin inhibits monocytes differentiate into macrophages through the AMPK-STATA3 pathway [67]. Li et al. [68] found that metformin can upregulate the expression of ABCG1 in murine macrophages. Our previous study also confirmed that metformin could attenuate atherosclerosis by increasing the cholesterol efflux capacity of macrophages [33]. We also hypothesize that metformin may promote cholesterol efflux in macrophages by up-regulating FGF21 expression [69].

## Conclusion

Metformin has been widely used as an anti-diabetic drug to treat patients with DM. Its cardioprotective role is being increasingly realized beyond its glucose lowering effect, although there may be some overlap of these two properties in the systemic cardioprotective effects. However, the anti-atherosclerotic effects of metformin, independent of its glycemic control remain unclear and is an area of active research. Evidence backing the pleiotropic effects of metformin in reducing CVD-related events necessitates further exploration with regard to the mechanism of drug action in various tissue compartments. The recent evidence pointing towards its therapeutic benefit in heart failure with persevered ejection fraction in clinical and preclinical studies underlines the need to explore the breadth of the pleiotropic systemic effects of metformin [70]. Recent study also reported that metformin offers therapeutic benefit during heart failure with preserved ejection fraction by lowering titin-based passive stiffness in mice model [71], is associated with improved survival and decreased incidence of adverse cardiac events in peripheral arterial disease patients [72] and with a lower below-the-knee arterial calcification score [73]. Moreover, metformin is able to prevent cardiac dysfunction in a murine model of adult congenital heart disease [70]. A proper understanding of these pleotropic effects will allow us to tailor the dose of the drug and remain abreast about its potential side effects in patients receiving them. Apart from their use in DM patients, its role in primary prevention of cardiovascular outcomes in ‘at-risk’ population, akin to statins, requires further exploration. Prospective population studies and randomized clinical trials need to be conducted to identify will allow us to identify a subset of patients who may benefit from early administration of metformin. This will further aid in disease surveillance and intervention through enhancement of primary and secondary prevention of CVD.

## Abbreviations

ABCA1: ATP-binding cassette transporter A1
ASCVD: atherosclerotic cardiovascular disease
CAD: coronary artery disease
CAMERA: Carotid Atherosclerosis: MEtformin for insulin ResistAnce
DM: diabetes mellitus
MACE: major adverse cardiovascular events
PTCA: percutaneous transluminal coronary angioplasty
REMOVAL: REversing with MetfOrmin Vascular Adverse Lesions
SPREAD-DIMCAD: study on the prognosis and effect of antidiabetic drugs on type 2 diabetes mellitus with coronary artery disease
TC: total cholesterol
T2DM: type 2 diabetes mellitus
TG: triglyceride
VLDL: very low-density lipoprotein
CIMT: carotid intima-media thickness
UKPDS: UK Protective Diabetes Study
VSMCs: vascular smooth muscle cells
